# On the Optimization of the Protocol for Automated Radiosyntheses of [^68^Ga]Ga-Pentixafor, [^68^Ga]Ga-FAPI-4 and [^68^Ga]Ga-DOTATATE in a Modular-Lab Standard

**DOI:** 10.22038/AOJNMB.2024.77059.1545

**Published:** 2024

**Authors:** Sreeja Raj Menon, Arpit Mitra, Sudeep Sahu, Sangita Lad, Avik Chakraborty, Mukti Kanta Ray, Sharmila Banerjee

**Affiliations:** 1 Health Physics Division, Bhabha Atomic Research Centre, Mumbai, India; 2 Homi Bhabha National Institute, Mumbai, India; 3 Radiopharmaceutical Laboratory, Board of Radiation and Isotope Technology, Navi Mumbai, India; 4 Radiation Medicine Centre, Bhabha Atomic Research Centre, Mumbai, India; 5 Advanced Centre for Treatment, Research and Education in Cancer, Tata Memorial Centre, Navi Mumbai, India

**Keywords:** [^68^Ga]Ga-Pentixafor, [^68^Ga]Ga-FAPI-4, [^68^Ga]Ga-DOTATATE, Modular-Lab Standard, PET/CT

## Abstract

**Objective(s):**

The present work describes the automated radiochemical synthesis of different PET tracers like [^68^Ga]Ga-Pentixafor, [^68^Ga]Ga-FAPI-4 and [^68^Ga]Ga-DOTATATE using optimized single protocol in the non-cassette based Eckert & Ziegler (EZ) Modular Lab (fixed tubing system) without any modification in the inbuilt human machine interface (HMI) software. Recently, PET agents viz. [^68^Ga]Ga-Pentixafor and [^68^Ga]Ga-FAPI-4 are gaining prominence for the diagnosis of overexpressed Chemokine Receptor-4 (CXCR4) and Fibroblast Activation Protein (FAP) receptor, respectively, in the microenvironment of numerous cancer types. The promising results observed with the clinical usage of [^68^Ga]Ga-DOTATATE produced using the automated protocol, provided impetus for the clinical translation of [^68^Ga]Ga-Pentixafor and [^68^Ga]Ga-FAPI-4 using the in-house developed automated radiolabeling protocol.

**Methods:**

Herein we report a single radiolabeling protocol for the automated preparation of [^68^Ga]Ga-Pentixafor and [^68^Ga]Ga-FAPI-4 in the non-cassette based EZ Modular-Lab Standard radiochemistry module, without any changes in schematic, graphical user interface (GUI) software and time list, from that used for routine production of [^68^Ga]Ga-DOTATATE in our centre, since 2015. Physico-chemical quality control and in-vitro stability analyses were carried out using radio-TLC and radio-HPLC.

**Results:**

The automated protocol yielded reliable and consistent non-decay corrected (ndc) radiochemical yield (RCY) of (84.4%±0.9%) and (85.5%±1.4%) respectively, for [^68^Ga]Ga-Pentixafor and [^68^Ga]Ga-FAPI-4, with RCP>98%, which are comparable to the RCY of (84.4%±1.2%) and RCP (99.1%±0.3%) for [^68^Ga]Ga-DOTATATE. The biological quality control studies confirmed the formulations to be of ready-to-use pharmaceutical grade.

**Conclusion:**

The consistent and reliable RCY and RCP of multiple ^68^Ga-labeled PET tracers by single automated radiochemistry protocol exhibits the versatility of the EZ Modular Lab.

## Introduction

 PET/CT scintigraphy using [^68^Ga]Ga-labeled agents has gained prominence in Nuclear Medicine practices for the diagnosis and staging of different primary and malignant diseases (1). 

 Many newly developed ^68^Ga-based radiotracers also have earned importance as primary radiotracers for the detection of various non-malignant diseases (2). More recently, some of these ^68^Ga-based radiotracers have been used as theranostic pairs with either ^177^Lu or ^90^Y labeled radiopharmaceuticals (3). 

^18^F based radio-tracers are still considered to be the workhorse of PET radiopharmaceuticals, due to its (i) physical half-life of 109.7 minutes and (ii) capability of its production in multicurie level (4). However, the significant drawback of ^18^F-based radiotracers is the non-specific uptake (5). With the increasing clinical demand of ^68^Ga based radiotracers, the most critical aspect of concern is to ensure a consistent supply of an aseptic radiochemical formulation of these radiotracers in a hospital radiopharmacy setting. Automation of the radiolabeling process for ^68^Ga-labeled radiotracer preparations is an essential requirement to ensure compliance with the regulatory requirement for clinical use of positron emission tomography (PET) radiopharmaceuticals, with respect to radiation protection aspects, current Good Manufacturing Practices (cGMP) and Good Laboratory Practices (GLP) while conceiving this in a hospital radiopharmacy setting (6). The development of the automated radiochemistry module for radiosynthesis of ^68^Ga-based radiopharmaceuticals are based on aspects such as (i) post processing approach, (ii) system engineering and (iii) fluid path. In the current scenario, most of the commercial automated radiochemistry modules are designed based on the use of disposable strong cation exchange cartridge (post processing approach), (ii) dedicated compact system (system engineering) and (iii) disposable sterile cassette (fluid path)(4,7). 

 Nuclear Medicine Facilities equipped with therapy wards for housing of patients, have a high demand of various types of ^68^Ga-labeled radiopharmaceuticals, for staging various malignancies prior to carrying out radionuclide therapy. Towards planning radionuclide therapy (RNT) using therapeutic radio-conjugates Lutetium-177 or Yttrium-90 labeled Pentixather or FAPI-4, staging is an important requirement and is carried out using theranostic counterpart PET radiotracers [^68^Ga]Ga-Pentixafor and [^68^Ga]Ga-FAPI-4. This requires regular preparation of ^68^Ga labeled radiopharmaceuticals using existing automated radiochemistry module in the hospital radiopharmacy facility. Towards this, for our facility, the preparation of patient doses of [^68^Ga]Ga-DOTATATE, [^68^Ga]Ga-Pentixafor and [^68^Ga]Ga-FAPI-4 have been carried out using the automated EZ Modular-Lab Standard non-cassette based system. The strategy involves optimization of a single radiolabeling protocol to be used in the automated EZ Modular-Lab Standard, without any modification in the time list of in-built GUI software. The single radiolabeling protocol for the automated radiochemical synthesis of [^68^Ga]Ga-DOTATATE, [^68^Ga]Ga-Pentixafor and [^68^Ga]Ga-FAPI-4 has been rigorously optimized and the developed protocol was found to be efficient, consistent, reliable and reproducible. The produced [^68^Ga]Ga-DOTATATE, [^68^Ga]Ga-Pentixafor and [^68^Ga]Ga-FAPI-4 were in acceptable radiochemical yield (RCY), radiochemical purity (RCP), chemical purity (CP), solvent levels (SL), radioactive concentration (RAC) and endotoxin limit (EL). 

 The excellent localization of [^68^Ga]Ga-DOTATATE in somatostatin receptor avid liver and rectal lesions of a patient, while prominent localization of [^68^Ga]Ga-FAPI-4 in the skull base in classical case of adenoid cystic carcinoma (ACC) patients has demonstrated the clinical efficacy of [^68^Ga]Ga-DOTATATE and [^68^Ga]Ga-FAPI-4 synthesized in the EZ Modular-Lab Standard.

## Methods

***Reagents & Apparatus***

 [^68^Ga]GaCl_3_ was sourced from a ^68^Ge/^68^Ga generator (Matrix: SiO_2_, [^68^Ge]Ge^4+^ breakthrough: ≤0.005% of total radioactivity), which was obtained from ITM Medical Isotopes GmbH, Germany. Automated radiosynthesis module (Modular-Lab Standard) was from Eckert & Ziegler, Germany. The BFCA-Peptide conjugates viz. DOTA-TATE, DOTA-Pentixafor and DOTA-FAPI-4 were procured from PiChem (Austria), Lympholucin (South Korea) and MedChem Express (USA) respectively. HCl (30%, Ultrapur^®^), Sodium acetate trihydrate (BioUltra), Acetic acid (trace metal basis), NaCl (trace metal basis), Acetone (puriss) and Ethanol (Emsure) were procured from Merck, Germany. Ultrapure water (Trace SELECT^®^) was from Honeywell Research Chemicals, USA. 

 Disposable cation exchange resin viz. Strata™ XC and Strata™ SCX were obtained from Phenomenex, USA. Solid phase extraction (SPE) cartridges viz. plus C18 (360 mg), light C18 (130 mg) and light tC18 (145 mg) were procured from Waters Corporation, USA. Nitrogen (air purity: 99.999%) was purchased from Inox Air Products, India. Polyethersulfone (PES, pore size: 0.22 µm) and hydrophobic repel stripe membrane syringe filters were procured from Merck, Germany and Pall Corporation, USA, respectively. Non-bleeding type pH indicator strips (range: 0-14) were from Merck, India. 

 Sterile, pyrogen-free saline was procured from Nirlife Healthcare, India. Bio-safety cabinet (ISO Class 5) was purchased from Microfilt, India. 

 Analytical radio-high performance liquid chromatography (radio-HPLC) was performed using HPLC system from Knauer, Germany, equipped with UV and radioactive detectors, connected in series. Radio-thin layer chromatography (radio-TLC) was performed using miniGita equipped with NaI(Tl) radioactive detector from Bioscan, USA. 

 Radionuclide purity (RNP) was determined by recording gamma ray using high-purity germanium (HPGe) detector (Baltic Scientific Instruments, Russia), coupled to 64k multi-channel analyzer (MCA) (ITECH instruments, France).Gas chromatography was carried out in Chemito 7610 GC instrument from USA equipped with split/splitless injector inlet, flame ionization and thermal conductivity detector using helium as carrier gas.Dose calibrator (CRC 25R) was from Capintec, USA.Endotoxin limit (EL) was quantified by gel-clot BET assay method using LAL reagent from Charles River Laboratories Inc, USA, while the sterility test was performed by direct inoculation method using soyabean casein digest and fluid thioglycolate media from Himedia, India. PET/CT scintigraphy was carried out using Gemini Digital PET/CT scanner from Philips N.V, Netherlands.

***Preparation of Reagents and Preconditioning of SPE Cartridges for Radiochemical Synthesis***

 All reagents for automated radiosynthesis of [^68^Ga]Ga-Pentixafor, [^68^Ga]Ga-FAPI-4 and [^68^Ga] Ga-DOTATATE were prepared under aseptic conditions in bio-safety cabinet (ISO Class 5 grade). 

**i.** 0.05M HCl (for elution of [^68^Ga]Ga^3+^ from ^68^Ge/^68^Ga generator ) was prepared by aseptically mixing 0.53 mL of 30% HCl (Ultrapur^®^ grade, Purity: ≥99.99% trace metal basis) in 99.47 mL of ultrapure water (Trace SELECT^®^) and filtered through 0.22 µm sterile PES membrane syringe filter.

**ii.** Acidified NaCl (0.2N HCl in 5M NaCl, for pre-concentration and elution of [^68^Ga]GaCl_3_ from Strata™ SCX cartridge) was prepared by mixing 250 µL of 30% HCl (Suprapur^®^ grade, Purity: ≥99.99% trace metal basis) in 10 mL of 5M NaCl and filtered through 0.22 µm sterile PES membrane syringe filter.

**iii.** 1M CH3COONa buffer (reaction medium) was prepared by aseptically mixing 3.6 mL of 1M CH3COONa solution with 16.4 mL of 1M CH3COOH. The pH of the resultant 1M CH3COONa buffer was maintained between 3.5- 4.0 and filtered through 0.22 µm sterile PES membrane syringe filter.

**iv.** Stock solutions of DOTA-Pentixafor, DOTA-FAPI-4 and DOTA-TATE were prepared aseptically by separately dissolving 1 mg each of the lyophilized ligand or peptide (powder form) in 1 mL of ultrapure water (Trace SELECT®) and aliquoted in volume of 60 µL and stored at -20^o^C.

**v.** Each of the SPE cartridges (plus C18, light C18and light tC18) were preconditioned with 5 mL of ethanol (Emsure grade). Further, each of these cartridges were dried with 15 mL of air after 10 minutes post-conditioning with ethanol. Finally, 5 mL of ultrapure water (Trace SELECT®) was passed through each of these cartridges, followed by drying with 25 mL of air.

***Automated Radiosynthesis of [*** ^68^ ***Ga]Ga-Pentixafor, [*** ^68^ ***Ga]Ga-FAPI-4 and [*** ^68^ ***Ga]Ga-DOTATATE***

**Figure 1:**
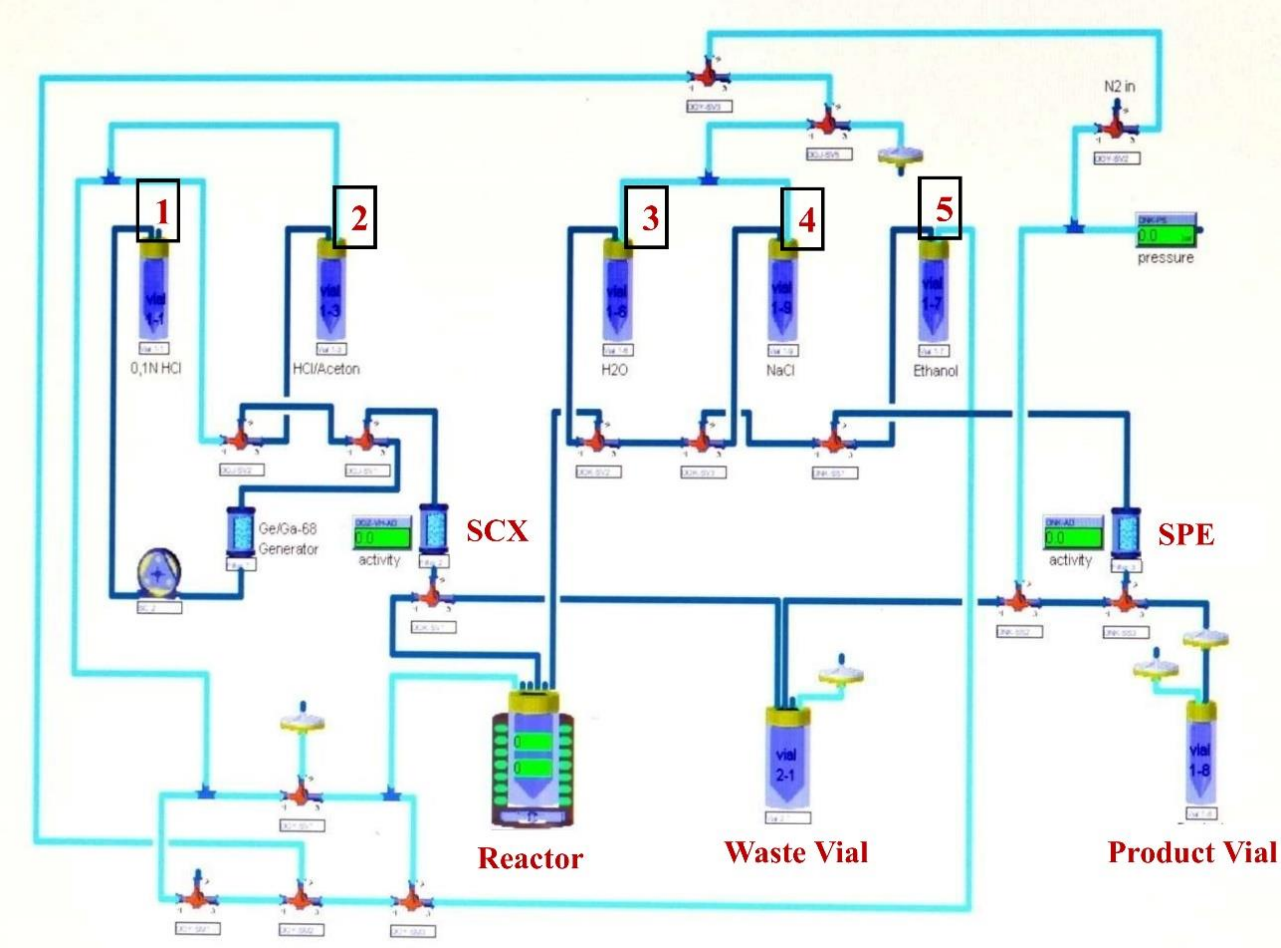
Schematics of EZ Modular Lab Standard for the Production of [^68^Ga]Ga-Pentixafor, [^68^Ga]Ga-FAPI-4 & [^68^Ga]Ga-DOTATATE

 Eckert & Ziegler (EZ) Modular-Lab Standard automated radiochemistry module, used for the radiosynthesis of [^68^Ga]Ga-Pentixafor, [^68^Ga] Ga-FAPI-4 and [^68^Ga]Ga-DOTATATE is shown in the Figure 1. The automated radiosynthesis for all the three radiotracers were carried out separately without any modification in the radiochemistry module. However, prior to automated radiosynthesis of each of these radiotracers ([^68^Ga]Ga-Pentixafor, [^68^Ga]Ga-FAPI-4 and [^68^Ga]Ga-DOTATATE) in the EZ Modular-Lab Standard, an automated cleaning or sanitization process of the EZ Modular-Lab Standard with 70% aqueous ethanol was mandatory. The automated protocol for radiolabeling Pentixafor, FAPI-4 and DOTATATE with [^68^Ga]Ga^3+^are depicted in Table 1. It is noteworthy that Step-1 and Step-2 were common for the radiosynthesis of each of these radiotracers ([^68^Ga]Ga-Pentixafor, [^68^Ga]Ga-FAPI-4 and [^68^Ga]Ga-DOTATATE) in the EZ Modular-Lab Standard. However, in the Step-3, different SPE cartridges have been used for post radiolabeling purification.

*Step 1: Elution and Pre-concentration of [* ^68^ *Ga]GaCl*_3_

(**i**) [^68^Ga]Ga^3+^ (1.11 GBq) in the form of [^68^Ga]GaCl_3_ was eluted from the ^68^Ge/^68^Ga generator with 4-5 mL of 0.05N HCl (Suprapur^®^ grade) and passed through Strata SCX (strong cation exchanger) column.

(**ii**) In Strata SCX column, [^68^Ga]Ga^3+^remained trapped, while [^68^Ge]Ge^3+^ (if any) and other nonradioactive metallic impurities were washed out from the column and collected in the waste vial.

(**iii**) Trapped [^68^Ga]Ga^3+^ in strata SCX column 

was pre-concentrated with 512µL freshly prepared acidified NaCl (0.2N HCl in 5M NaCl) and eluted in to the reaction vessel.

*Step 2: Radio Complexation*

(**i**) Radiolabeling of DOTA-Pentixafor: Pre- concentrated [^68^Ga]GaCl_3_ (512 µL, ~1.11 GBq) was mixed with 2.0mL of 1M CH_3_COONa buffer, containing 30 µL of DOTA-Pentixafor (30 µg, 24.6 nmoles).

 Radiolabeling of DOTA-FAPI-4: Pre-concentrated [^68^Ga]GaCl_3_ (512 µL, ~1.11 GBq) was mixed with 2.0 mL of 1M CH_3_COONa buffer containing 30 µL of DOTA-FAPI-4 (30 µg, 34.4 nmoles).

 Radiolabeling of DOTA-TATE: Pre-concentrated [^68^Ga]GaCl_3_ (512 µL, ~1.11 GBq) was mixed with 2.0 mL of 1M CH_3_COONa buffer containing 30 µL of DOTA-TATE (30 µg, 20.9 nmoles).

(**ii**) Each of these reaction mixtures were incubated at 95^o^C for 8 minutes at pH~4.0 and thereafter cooled to room temperature (25^o^C) by adding 2.0 mL of ultrapure water.

*Step 3: Purification through SPE Cartridge post-radiolabeling*

(**i**) [^68^Ga]Ga-Pentixafor, [^68^Ga]Ga-FAPI-4 and [^68^Ga]Ga-DOTATATE reaction mixtures were loaded on to preconditioned plus C18, light C18 and light tC18 cartridges respectively. Each of these eluates was sent to a waste vial.

(**ii**) plus C18, light C18 and light tC18 cartridges loaded with [^68^Ga]Ga-Pentixafor, [^68^Ga]Ga-FAPI-4 and [^68^Ga]Ga-DOTATATE reaction mixtures were rinsed with 2 mL of sterile pyrogen free saline respectively to remove unlabeled free [^68^Ga]Ga^3^^+^.

(**iii**) The purified products [^68^Ga]Ga-Pentixafor (~0.94 GBq), [^68^Ga]Ga-FAPI-4 (~0.94 GBq) and [^68^Ga]Ga-DOTATATE(~0.92 GBq) were eluted from plus C18, light C18 and light tC18 cartridge respectively using 0.8 mL of 50% aqueous ethanol, through 0.20 μm PES membrane syringe filter into the final product vials.

(**iv**) Post elution of each of the product with aqueous ethanol (50%), sterile pyrogen free saline (8 mL) was passed through the SPE cartridges and 0.20 μm PES membrane filter, for dilution of the final product, so as the RAC for each of the product was maintained between 0.08-0.11 GBq/mL.

**Table 1:** Comparative Positions of Reservoirs for Chemicals used in the EZ Modular Lab Standard for Radiosynthesis of [^68^Ga]Ga-Petixafor, [^68^Ga]Ga-FAPI-4 and [^68^Ga]Ga-DOTATATE

**Reservoir Number/ Position**	**[** ^68^ **Ga]Ga-Pentixafor**	**[** ^68^ **Ga]Ga-FAPI-4**	**[** ^68^ **Ga]Ga-DOTATATE**
Reservoir-1	0.05M HCl, 4 mL - for eluting [^68^Ga]Ga^3+^ from ^68^Ge/^68^Ga generator	0.05M HCl, 4 mL - for eluting [^68^Ga]Ga^3+^ from ^68^Ge/^68^Ga generator	0.05M HCl, 4 mL - for eluting [^68^Ga]Ga^3+^ from ^68^Ge/^68^Ga generator
Reservoir-2	Acidified NaCl, 512 µL (0.2M HCl in 5M NaCl) - for pre-concentrating [^68^Ga]GaCl_3_	Acidified NaCl, 512 µL (0.2M HCl in 5M NaCl) - for pre-concentrating [^68^Ga]GaCl_3_	Acidified NaCl, 512 µL (0.2M HCl in 5M NaCl) - for pre-concentrating [^68^Ga]GaCl_3_
Reservoir-3	Ultrapure H_2_O (2 mL) - for cooling the reaction mixture	Ultrapure H_2_O (2 mL) - for cooling the reaction mixture	Ultrapure H_2_O (2 mL) - for cooling the reaction mixture
Reservoir-4	0.9% NaCl (10 mL) – (i) 2 mL for removing free [^68^Ga]Ga^3+^ from plus C18 column & (ii) 8 mL for dilution of final product	0.9% NaCl (10 mL) – (i) 2 mL for removing free [^68^Ga]Ga^3+^ from light C18 column & (ii) 8 mL for dilution of final product	0.9% NaCl (10 mL) – (i) 2 mL for removing free [^68^Ga]Ga^3+^ from light tC18 column & (ii) 8 mL for dilution of final product
Reservoir-5	50% aqueous C_2_H_5_OH (0.8 mL) - for eluting [^68^Ga]Ga-Pentixafor from plus C18 column	50% aqueous C_2_H_5_OH (0.8 mL) - for eluting [^68^Ga]Ga-FAPI-4 from light C18 column	50% aqueous C_2_H_5_OH (0.8 mL) - for eluting [^68^Ga]Ga-DOTATATE from light tC18 column
Reactor	DOTA-Pentixafor (30 µg / 30 µL, 24.6 nmoles) in 2 mL of 1M CH_3_COONa buffer (pH: ~4.0)	DOTA-FAPI-4 (30 µg / 30 µL, 34.7 nmoles) in 2 mL of 1M CH_3_COONa buffer (pH: ~4.0)	DOTATATE (30 µg / 30 µL, 20.9 nmoles) in 2 mL of 1M CH_3_COONa buffer (pH: ~4.0)
Product Vial	Nil	Nil	Nil
SPE Cartridge used post-radiolabeling	Plus C18 (360 mg)	Light C18 (130 mg)	Light tC18 (145 mg)

***Quality Control of [*** ^68^ ***Ga]Ga-Pentixafor, [*** ^68^ ***Ga]Ga-FAPI-4 and [*** ^68^ ***Ga]Ga-DOTATATE***

 The pH of all the three ^68^Ga-labeled radiotracers were measured by observing the color change of the narrow range pH strips after spotting 1-2 µL of the final product. The RCP of all the three [^68^Ga] radiotracers were assessed by radio-TLC (silica gel plastic TLC plates, pore size: 60Ao) using 0.1M sodium citrate buffer (pH: 5.0) as eluant. The RCP of all the three [^68^Ga] radiotracerswas also ascertained by radio-HPLC using Eurosphere C-18 Reversed phase column {Dimension: 300 mm (Length) × 4 mm (Diameter), Particle size: 5µm} coupled with NaI(Tl) and UV detector maintaining a flow rate of 1.0 mL/min in gradient mode. The gradient method for HPLC analysis of [^68^Ga]Ga-Pentixafor, [^68^Ga]Ga-FAPI-4 and [^68^Ga]Ga-DOTATATE were specific for each of these radiotracers. The solvent system was common for all the three ^68^Ga radiotracers (water and acetonitrile containing 0.1% trifluoro acetic acid), The gradient mode of elution was specific for each of the product viz. [^68^Ga]Ga-Pentixafor (0–20 min 90% to 30% water, 20–21 min 30% to 0% water, 21–30 min 0% water and 30 – 40 min 0% to 100% water), [^68^Ga]Ga-FAPI-4 (0–2 min 95% water, 2–10 min 95% to 50% water, 10–12 min 50% water 12 -14 min 50% to 100% water and 14–20 min 100% water) and [^68^Ga]Ga-DOTATATE (0–5 min 95% water, 5 – 10 min 95% to 60% water, 10 –11 min 60% to 95% water and 11 - 25 min 95% water). While doing HPLC of [^68^Ga]-Pentixafor, UV wavelength was set at 280 nm. On the other hand, for [^68^Ga]Ga-FAPI-4 and [^68^Ga]Ga-DOTATATE, UV wavelength were set at 220 nm. 

 For all the three ^68^Ga radiotracers, the ethanol levels in the final product were detected by gas chromatography using a standardized procedure, with helium as carrier gas and polyethylene glycol capillary column {Dimension: 25 m (L) × 0.32 mm (ID), Thickness: 0.5 µm}. The capillary column was maintained at a temperature of 70^o^C during the operation. The endotoxin limit (EL) was quantified by gel-clot BET assay method using lysate with sensitivity 0.125 EU/mL, at 200 maximum valid dilution (MVD). Sterility test was carried out by direct inoculation method. 

 In-vitro stability of [^68^Ga]Ga-Pentixafor, [^68^Ga]Ga-FAPI-4 and [^68^Ga]Ga-DOTATATE were evaluated by radio-HPLC, at 4h post-radiolabeling. In brief, all the three ^68^Ga radiotracers (each with 0.8 mL volume) were incubated with 1.2 mL of saline at room temperature (25^o^C) for 4h, further radio-HPLC were carried out post 4h of incubation for all the three radiotracers separately.

***Regulatory Approval***

 The EZ Modular-Lab Standard along with ^68^Ge/^68^Ga generator was procured by our facility in the year 2015 and the automated system is being used routinely for the preparation of [^68^Ga]Ga-DOTATATE for patients use, after obtaining regulatory approval from the Radiopharmaceutical Committee (RPC) and the BARC Safety Council (BSC) for clinical use in patients and radiological safety of operation respectively. Presently, the same EZ Modular-Lab Standard have been used for automated radiochemical synthesis of two other different ^68^Ga based radiotracers namely [^68^Ga]Ga-Pentixafor and [^68^Ga]Ga-FAPI-4. While the regulatory approval for routine clinical use of [^68^Ga]Ga-FAPI-4 using EZ Modular-Lab Standard has been obtained, the regulatory clearance for clinical use of [^68^Ga]Ga-Pentixafor using the EZ Modular-Lab Standard is in the final stage.

***Clinical Studies of [*** ^68^ ***Ga]Ga-DOTATATE and [*** ^68^ ***Ga]Ga-FAPI-4 in Patients: Methodological Considerations***

 Multiple patient doses (~0.92 GBq) of [^68^Ga]Ga-DOTATATE were formulated per batch, using EZ Modular-Lab Standard. From the preparation, diagnostic doses of [^68^Ga]Ga-DOTATATE (~ 0.14 GBq per patient) were routinely administered in patients, with known cases of neuroendocrine tumor (NET). The diagnostic efficacy of [^68^Ga]Ga-DOTATATE was evaluated in NET patients in 504 different batches, since 2015. The clinical PET/CT imaging studies of NET patients injected with [^68^Ga]Ga-DOTATATE were carried out using a dedicated Gemini PET/CT scanner from Philips.

 Apart from [^68^Ga]Ga-DOTATATE, multiple patient doses (~0.94 GBq) of [^68^Ga]Ga-FAPI-4 were formulated per batch, using the same EZ Modular-Lab Standard. From the preparation, diagnostic doses (~0.14 GBq per patient) were administered in patient, with classical case of Adenoid Cystic Carcinoma (ACC). The clinical SPECT/CT imaging studies of ACC patient injected with [^68^Ga]Ga-FAPI-4 were carried out using a dedicated PET/CT scanner.

***Preclinical Studies of [*** ^68^ ***Ga]Ga-Pentixafor in Tumor Bearing Mice***

 Receptor specific uptake of [^68^Ga]Ga-Pentixafor was studied in C57BL/6 mice bearing melanoma tumor xenografts (n=3). The CXCR4 receptors are reported to be overexpressed and active in metastatic melanoma, contributing towards cell proliferation and survival (8). Also, CXCR4/CXCL12 pathway regulates CXCL12 directed migration of tumor cells from their primary location to secondary sites (9). For inducing tumor in the mice, B_16_-F_10_ cell lines expressing CXCR4 receptor was used (10-12). 

 Mice were injected subcutaneously with melanoma cells (1×10^6^ cells per mouse) at the proximal flank area for tumor induction. Tumor was allowed to grow to about 1cm^3^ volume. The radiolabeled agent [^68^Ga]Ga-Pentixafor was injected to tumor bearing mice (~7.4 MBq) through the tail vein. PET/CT imaging of mice was carried out at 1 h post-injection, followed by biodistribution studies.

## Results

***Automated Radiosynthesis of [*** ^68^ ***Ga]Ga-Pentixafor, [*** ^68^ ***Ga]Ga-FAPI-4 and [*** ^68^ ***Ga]Ga-DOTATATE***

 Using [^68^Ga]GaCl_3_ from SiO2 based ^68^Ge/^68^Ga generator, a fully automated radiochemical synthesis of [^68^Ga]Ga-Pentixafor (n=12), [^68^Ga]Ga-FAPI-4 (n=9) and [^68^Ga]Ga-DOTATATE (n=504) were carried out separately in a EZ Modular-Lab Standard. The radiochemical synthesis time for each of these ^68^Ga-labeled radiotracers was 15±2 minutes. 

 The non-decay corrected (ndc) RCY for [^68^Ga]Ga-Pentixafor, [^68^Ga]Ga-FAPI-4 and [^68^Ga]Ga-DOTATATE were found to be (84.4±0.9)% (n=12), (85.5±1.4)% (n=9) and (84.4±1.2)% (n=504) respectively.

***Quality Control of [*** ^68^ ***Ga]Ga-Pentixafor, [*** ^68^ ***Ga]Ga-FAPI-4 and [*** ^68^ ***Ga]Ga-DOTATATE***

**Figure 2:**
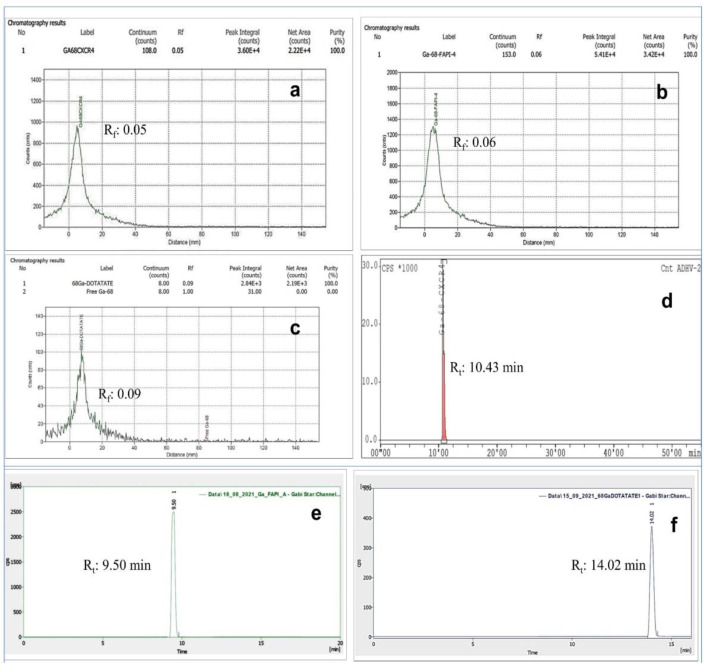
Radio-TLC chromatogram (in 0.1M Sodium Citrate Buffer (pH~5.0) as eluant) of (**a**) [^68^Ga]Ga-Pentixafor (Rf: 0.05), (**b**) [^68^Ga]Ga-FAPI-4 (Rf: 0.05), (**c**) [^68^Ga]Ga-DOTATATE (Rf: 0.05), Radio-HPLC chromatogram of (**d**) [^68^Ga]Ga-Pentixafor (Rt: 10.43 min), (**e**) [^68^Ga]Ga-FAPI-4 (Rt: 9.50 min) and (**f**) [^68^Ga]Ga-DOTATATE (Rt: 14.02 min)

 The products [^68^Ga]Ga-Pentixafor (n=12), [^68^Ga]Ga-FAPI-4 (n=9) and [^68^Ga]Ga-DOTATATE (n=504) were found to be clear and colorless. The pH of all the three radiotracers were ~4.5, while the RAC were between 0.08-0.11GBq/mL. The RCP of [^68^Ga]Ga-Pentixafor (n=12), [^68^Ga]Ga-FAPI-4 (n=9) and [^68^Ga]Ga-DOTATATE (n=504), assessed by radio-TLC were (98.7±0.3)%, (98.9±0.2)% and(98.7±0.3) % respectively. In 0.1 M sodium citrate buffer (pH: 5.0) solvent system radio-TLC, the observed Rf values were 0.05 for [^68^Ga]Ga-Pentixafor (Figure 2a), 0.06 for [^68^Ga]Ga-FAPI-4 (Figure 2b) and 0.09 for [^68^Ga]Ga-DOTATATE (Figure 2c). The RCP of [^68^Ga]Ga-Pentixafor (n=12), [^68^Ga]Ga-FAPI-4 (n=9) and [^68^Ga]Ga-DOTATATE (n=504) were also evaluated by radio-HPLC and were found to be (99.4±0.3)% (n=12), (99.6±0.2)% (n=9) and (99.1± 0.3)% (n=504) with Rt values10.4 minutes (Figure 2d), 9.5 minutes (Figure 2e) and 14.0 minutes (Figure 2f) respectively.

 The in-vitro stability of the final product in saline (without stabilizer) post 4 h of radiolabeling, at room temperature (25^o^C) has been determined by radio-HPLC. Retention of RCP (98.9%±0.4%) was observed for [^68^Ga]Ga-Pentixafor (Rt: 10.5 minutes). Similarly, radio-HPLC chromatograms indicated the retention of RCP to the extent (98.9%±0.3%) for [^68^Ga]Ga-DOTATATE (Rt: 14.0 minutes) and (98.8%±0.2%) for [^68^Ga]Ga-FAPI-4 (Rt:9.4 minutes) respectively.

 The ethanol content in [^68^Ga]Ga-Pentixafor, [^68^Ga]Ga-FAPI-4 and [^68^Ga]Ga-DOTATATE were found to be (6.4%±0.2%), (6.4%±0.3%) and(6.3%±0.1%) respectively. The specific activities of the radiotracers [^68^Ga]Ga-Pentixafor, [^68^Ga]Ga-FAPI-4 and [^68^Ga]Ga-DOTATATE were evaluated to be (37.7±1.8) MBq/nmole,(38.1±1.5) MBq/nmole and (44.0±0.9) MBq/nmole respectively and the preparations were found to be sterile with the endotoxin limits being <25 EU/mL.

 The RNP derived by γ-spectroscopy for all the three [^68^Ga] radiotracers were found to be >99.9% with prominent 511 keV and 1077 keV  peak characteristics of [^68^Ga]Ga^3+^. The [^68^Ge]Ge^4+^ breakthrough was not estimated in the products, since the [^68^Ga]GaCl_3_used for radiolabeling was sourced from commercial ^68^Ge/^68^Ga generator. The quality control parameters of [^68^Ga]Ga-Pentixafor, [^68^Ga]Ga-FAPI-4 and [^68^Ga]Ga-DOTATATE using optimized single radiolabeling protocol in an automated radiochemistry module (EZ Modular-Lab Standard) were compared to that of specifications stated in the European Pharmacopoeia of Gallium (^68^Ga) Edotreotide^® ^injection ([^68^Ga]Ga-DOTA-TOC) as depicted in Table 2.

**Table 2:** Comparison of the produced [^68^Ga]Ga-Pentixafor, [^68^Ga]Ga-FAPI-4 and [^68^Ga]Ga-DOTATATE with that of [^68^Ga]Ga-Edotreotide^®^# as mentioned in Pharmaeuropa 23.2

**Description**	**Specifications of** **[** ^68^ **Ga]Ga-Edotreotide# (Pharmaeuropa 23.2)**	**Observed Results**		
		**[** ^68^ **Ga]Ga-Pentixafor**	**[** ^68^ **Ga]Ga-FAPI-4**	**[** ^68^ **Ga]Ga-DOTATATE**
Characteristics of the Solution	Clear & Colorless	Clear & Colorless	Clear & Colorless	Clear & Colorless
pH	4.0 – 8.0	~ 4.0 – 5.0	~ 4.0 – 5.0	~ 4.0 – 5.0
RAC	> 50 MBq/mL	~ 80-110 MBq / mL	~ 80-110 MBq / mL	~80-110 MBq / mL
RCY*	NA	84.4±0.9%	85.5±1.4%	84.4±1.2%
RNP	≥ 98 % as ^68^Ga^3+^	> 99%	> 99%	> 99%
RCP	≥ 95%	99.4±0.3%	99.6±0.2%	99.1±0.3%
Absolute Ethanol	< 10% V/V	6.4±0.2% (v/v)	6.4±0.3% (v/v)	6.3±0.1% (v/v)
CP (HEPES Content)	< 200 µg/V	NA	NA	NA
Endotoxin Limit	< 175 EU/V	< 25 EU/mL	< 25 EU/mL	< 25 EU/mL
Sterility Test (Post-facto)	Absence of any growth on 14 days of incubation	No growth	No growth	No growth
In-vitro Stability over 3h	≥ 95%	98.9±0.4%	98.8±0.2%	98.9±0.3%
Specific Activity	1 – 60 MBq/nmole	37.7±1.9 MBq/nmole	38.1±1.5 MBq/nmole	44.0± 0.9 MBq/nmole

RAC: Radioactive Concentration, RCY*: Radiochemical yield (Non Decay Corrected), RNP: Radionuclide purity, RCP: Radiochemical purity, CP: Chemical purity, HEPES: 2-[4-(2-hydroxyethyl)piperazin-1-yl]ethanesulfonic acid, #[^68^Ga]Ga-Edotreotide^®^: [^68^Ga]Ga-DOTA-TOC, (TOC: Tyrosine-3-octreotate)

***Clinical and Pre-clinical PET/CT imaging with [*** ^68^ ***Ga]Ga-labeled PET tracers***

***[*** ^68^ ***Ga]Ga-DOTATATE and [*** ^68^ ***Ga]Ga-FAPI-4 PET/CT Image of Patient***

**Figure 3:**
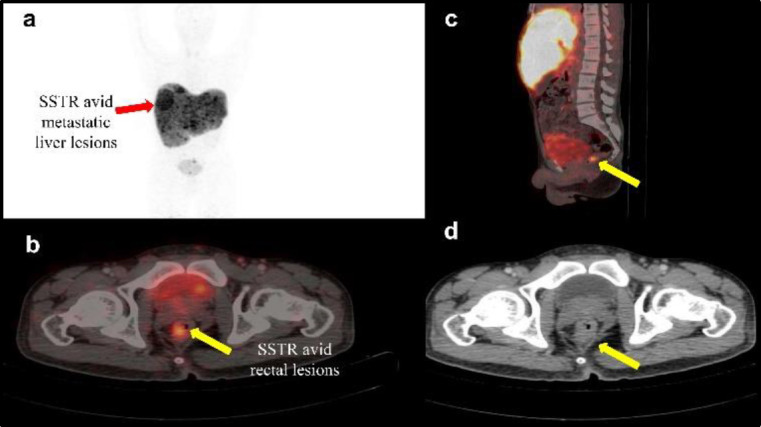
[^68^Ga]Ga-DOTATATE PET/CT Scan showing uptake in: (**a**) SSTR-avid metastatic liver lesions, (**b**) SSTR-avid rectal lesion, (**c**) SSTR-avid rectal lesion: sagittal fused image and (**d**) axial CT image

 The representative PET/CT image in a patient depicting the uptake and localization of [^68^Ga]Ga-DOTATATE, prepared using the optimized protocol, has been presented in Figure 3a-3c. The PET/CT image has been acquired 30 minutes post injection of 75 – 140 MBq of [^68^Ga]Ga-DOTATATE, in a patient with known case of grade I rectal NET (Ki index=1%), with metastatic liver disease showing evidence of metastatic liver and rectal lesions. The [^68^Ga]Ga-DOTATATE PET/CT scan of patient with maximum intensity projection exhibit somatostatin receptor (SSTR) avid metastatic liver lesions (Figure 3a). The trans axial and sagittal fused image of the [^68^Ga]Ga-DOTATATE PET/CT scan in the same patient exhibit SSTR-avid rectal lesions (Figure 3b) and metastatic liver lesions (Figure 3c) respectively. 

 The axial CT of [^68^Ga]Ga-DOTATATE PET/CT scan exhibit prominent rectal lesions (Figure 3d).

**Figure 4:**
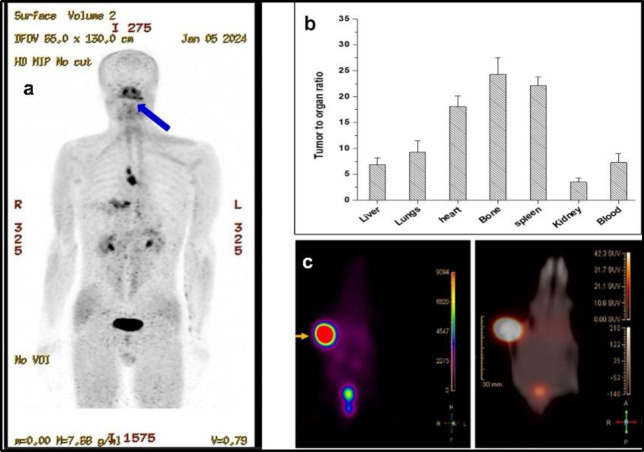
(**a**) [^68^Ga]Ga-FAPI-4 PET/CT Scan exhibiting intense uptake in lesions of skull base of Adenoid Cystic Carcinoma patient; (**b**) Tumor-to-organ ratio of [^68^Ga]Ga-Pentixafor in mice at 1h post-injection; (**c**) PET/CT image of melanoma tumor bearing mouse injected with [^68^Ga]Ga-Pentixafor at 1h post- injection

 Clinical use of [^68^Ga]Ga-FAPI-4 for tumor with FAP overexpression, has witnessed a tremendous growth, in last few years, in the staging of various types of cancers. The PET/CT image acquired 30 minutes post injection of 120 – 140 MBq of [^68^Ga]Ga-FAPI-4 in ACC patient with recurrence of lesions in skull base (Figure 4a) is an example demonstrating the clinical outcome of [^68^Ga]Ga-FAPI-4, thus produced in the EZ Modular-Lab Standard.

***[*** ^68^ ***Ga]Ga-Pentixafor PET/CT Image in mouse bearing melanoma tumor xenografts***

 Tumor uptake of (19.5%±2.5%) was observed at 1 h post-injection, with tumor-to-blood ratio of ~7.2. The tumour-to-organ ratios was greater than 3 for all the major organs, suggesting high contrast imaging (Figure 4b), which is in accordance with our PET/CT scintigraphy data (Figure 4c).

## Discussion

 In recent years, the automated radiosynthesis of [^68^Ga]Ga-Pentixafor using cassette based Modular Lab PharmTracer has been reported (13). Also, the automated synthesis of [^68^Ga]Ga-FAPI-46 using the radiochemistry module namely Trasis AiO Platform, GaSy Synthra, GallElut Scintomics, EazyOne Trasis, Modular Lab PharmTracer and Modular Lab easy has been reported (14-16). The factors common among these automated radiochemistry modules were (i) disposable cassette based (fluid path) and (ii) fully equipped compact system (system engineering). While these cassette-based and fully equipped compact system have advantages of (i) easy handling, (ii) devoid of cleaning and sanitization process prior radiolabeling (iii) better microbiological safety (iv) no cross-contamination and (iv) better cGMP and GLP compliance, they have several limitations also. These include (i) adaptability (i.e., requirement for development of different cassettes for different radiotracers), (ii) high cost of the cassette and (iii) malfunctioning problems (leakage, formation of bubbles etc.) during the radiosynthesis process (7).These limitations in cassette-based automated radiochemistry module poses operational challenges during the radiosynthesis of [^68^Ga]Ga-Pentixafor and [^68^Ga]Ga-FAPI-4,which emerges from the fact that we need to depend on the manufacturers for development of radiotracer-specific cassettes which, in turn, contributes to the high cost.

 To the best of our knowledge, automated radiochemical synthesis of [^68^Ga]Ga-Pentixafor and [^68^Ga]Ga-FAPI-4 have not yet been reported using any commercial modular fixed tubing system and the present work reported herein, on the automated radiochemical synthesis of [^68^Ga]Ga-Pentixafor and [^68^Ga]Ga-FAPI-4 in Modular-Lab Standard, which is routinely used for synthesis of [^68^Ga]Ga-DOTATATE and [^68^Ga]Ga-PSMA-11, constitutes the first of its kind. The automated radiochemistry module used by us is a modular (system engineering) and fixed tubing (fluid path) system. Hence the radiosynthesis of [^68^Ga]Ga-Pentixafor and [^68^Ga]Ga-FAPI-4 could be easily accomplished with only commercially available ligands and could obviate the need for development of radiotracer-specific cassettes by the manufacturer.

 A brief automated cleaning process of the EZ Modular-Lab Standard with 70% aqueous ethanol prior to radiolabeling of [^68^Ga]Ga-Pentixafor or [^68^Ga]Ga-FAPI-4 is required to prevent cross-contamination. Soon after the completion of radiolabeling procedure of each ^68^Ga radiotracer, the cartridges Strata™ XC/Strata™ SCX, plus C18/light C18/light tC18 and 0.22 µm membrane filter were removed, followed by purging the automated EZ Modular-Lab Standard with high purity nitrogen. Then automated cleaning process of EZ Modular-Lab Standard was carried out, by two times repeated cleaning, with 70% aqueous ethanol. 

 Radio-TLC and Radio-HPLC of aqueous ethanol (70%), collected post second time cleaning, have been carried out in the same solvent system used for the radioconjugate analysis. The radio-TLC and radio-HPLC chromatograms did not exhibit any significant radioactive peak, ensuring the absence of residual ^68^Ga radiotracers, prepared in the previous batch. This has been demonstrated successfully by consistent usage of the EZ Modular-Lab Standard for radiosynthesis of [^68^Ga]Ga-Pentixafor or [^68^Ga]Ga-FAPI-4, successively after routine radiosynthesis of [^68^Ga]Ga-DOTATATE or [^68^Ga]Ga-PSMA-11 on the same day, without any cross contamination.

 The EZ Modular-Lab Standard operates on GUI software. Hence temperature control, timer control, output pressure control for nitrogen air, flow rate and flow direction of solenoid valves could be modified in the program med time-list. In our optimized protocol for automated radiosynthesis of [^68^Ga]Ga-Pentixafor and [^68^Ga]Ga-FAPI-4, modification in programmed time-list was not required, since the radiotracers could be prepared with reasonably good non-decay corrected (ndc) RCY of (84.4%±0.9%) and (85.5%±1.4%) respectively. The ndc RCY and RCP of [^68^Ga]Ga-Pentixafor and [^68^Ga]Ga-FAPI-4 were comparable to that of the ndc RCY and RCP of [^68^Ga]Ga-DOTATATE.

**Figure 5:**
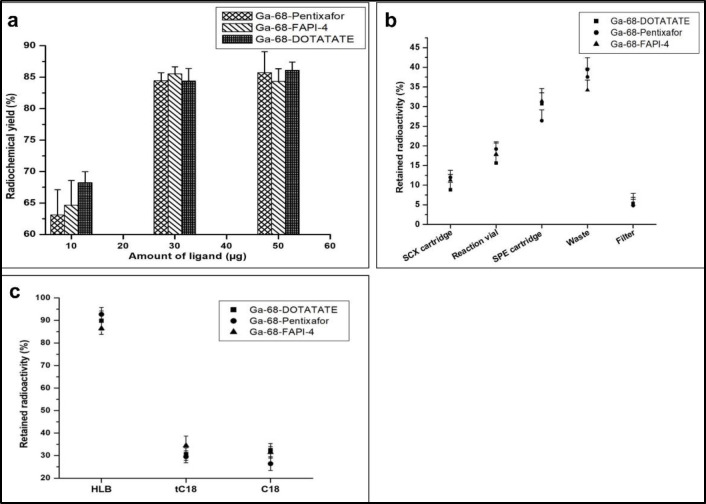
(**a**) Influence of ligand amount on RCY of [^68^Ga]Ga-Pentixafor, [^68^Ga]Ga-FAPI-4 & [^68^Ga]Ga-DOTATATE, (**b**) overall distribution of lost radioactivity in consumables of Modular-Lab Standard during production of [^68^Ga]Ga-Pentixafor , [^68^Ga]Ga-FAPI-4 & [^68^Ga]Ga-DOTATATE, (**c**) Retention of radioactivity in different SPE cartridges (HLB, tC18 & C18), during post-radiolabeling purification of [^68^Ga]Ga-Pentixafor , [^68^Ga]Ga-FAPI-4 & [^68^Ga]Ga-DOTATATE

^68^Ga-labeled radiopharmaceuticals demand high RCY, due to very short physical half-life of Gallim-68 (t1/2=67.8 minutes). Towards this, we carried out the automated radiosynthesis of [^68^Ga]Ga-Pentixafor and [^68^Ga]Ga-FAPI-4, using higher amounts (50 µg) of Pentixafor and FAPI-4 ligands to see whether the ndc RCY could be increased, in comparison to what is obtained when 30 µg is used in our optimized radiolabeling protocol. The results as depicted in Figure 5a indicate that there was no significant increase in ndc RCY for either of the products. In this automated synthesis of [^68^Ga]Ga-Pentixafor and [^68^Ga]Ga-FAPI-4, we have observed some loss of radioactivity. The results as depicted in Figure 5b indicate almost ~64-66% of lost radioactivity was in the SPE cartridges as well as in the waste washing vial, used for purification post-radiolabeling. The loss of radioactivity in the SPE cartridges (in case of [^68^Ga]Ga-Pentixafor and [^68^Ga]Ga-FAPI-4) were also comparable to the loss of radioactivity (~70%) as observed during [^68^Ga]Ga-DOTATATE radiosynthesis using the same system.

 Efforts were made to carry out the purification of both these radiotracers ([^68^Ga]Ga-Pentixafor and [^68^Ga]Ga-FAPI-4) with light tC18 instead of plus C18 and light C18 SPE cartridges. From Figure 5c, it was observed that there was no significant increase in the ndc RCY for both these radiotracers, since radioactivity retained in tC18 cartridges were almost equivalent to that of C18 cartridges, post elution with 0.8 mL of 50% aqueous ethanol. Also purification post-radiolabeling was carried out for [^68^Ga]Ga-Pentixafor and [^68^Ga]Ga-FAPI-4 with plus hydrophilic lipophilic balance (plus HLB) SPE cartridges [sorbent weight: 225 mg, particle size: 60 µm, pore size: 80A^o^, functional group: {poly(divinylbenzene-co-N-vinylpyrrolidone)}]. 

 We observed (Figure 5c) that the retained radioactivity of [^68^Ga]Ga-Pentixafor and [^68^Ga]Ga-FAPI-4 were ~92% and ~86% of lost radioactivity respectively, post-elution with 0.8 mL of 50% aqueous ethanol (incomplete elution from plus HLB column). However, on using 0.8 mL of 100% absolute ethanol as eluant for eluting [^68^Ga]Ga-Pentixafor and [^68^Ga]Ga-FAPI-4 from plus HLB cartridges, we observed that the percentage of lost radioactivity of [^68^Ga]Ga-Pentixafor and [^68^Ga]Ga-FAPI-4 retained in plus HLB cartridges were reduced to ~87% and ~82% respectively. Retention of both the radiolabeled products in plus HLB cartridges were higher compared to the C18 or tC18 cartridges, since 0.8 mL of 100% absolute ethanol was not sufficient in disrupting the Van der Waals forces existing between [^68^Ga]Ga-Pentixafor or [^68^Ga]Ga-FAPI-4 and the sorbent (HLB) (17-19).

## Conclusion

 A single radiolabeling protocol for automated radiosynthesis of [^68^Ga]Ga-Pentixafor and [^68^Ga]Ga-FAPI-4 has been established using a Modular-Lab Standard, with reproducible and consistent RCY and RCP. The automated radiolabeling protocol for these two radiotracers were similar to that of [^68^Ga]Ga-DOTATATE which is being routinely produced using the same automated module, thus establishing the robustness and versatility of the automated radiochemistry module. The excellent PET/CT images of NET and ACC patient injected with [^68^Ga]Ga-DOTATATE and [^68^Ga]Ga-FAPI-4 documents the quality of the produced products respectively. PET/CT image of CXCR4 expressing melanoma tumor bearing mice clearly delineated the tumor, with high contrast, at 1 h post-injection. These results demonstrate the merits of indigenously standardized single automated radiolabeling protocol for producing [^68^Ga]Ga-Pentixafor and [^68^Ga]Ga-FAPI-4 in the non-cassette based EZ Modular-Lab Standard. This development obviates the need for procuring commercially available cassettes which otherwise require optimization with respect to making it specific for a particular radiotracer as well as importing and marketing authorization. 

 The radiosynthetic strategy demonstrated herein, using the automated system, EZ Modular-Lab Standard, offers potential for preparation of other ^68^Ga-based PET radiotracers, for in house use.
